# Genetic variant of *miR-146a* rs2910164 C>G and gastric cancer susceptibility

**DOI:** 10.18632/oncotarget.8814

**Published:** 2016-04-18

**Authors:** Zu-Guang Xia, Hua-Fang Yin, Ying Long, Lei Cheng, Ling-Jun Yu, Wei-Jian Guo, Xiao-Dong Zhu, Jin Li, Ya-Nong Wang, Ya-Jun Yang, Jiu-Cun Wang, Li Jin, Li-Xin Qiu, Yongyue Wei

**Affiliations:** ^1^ Department of Medical Oncology, Fudan University Shanghai Cancer Center, Department of Oncology, Shanghai Medical College, Fudan University, Shanghai, China; ^2^ Department of Medical Oncology, The Affiliated Jiangyin Hospital of Southeast University Medical College, Jiangsu, China; ^3^ Department of Medical Oncology, Wanzai City Hospital, Jiangxi, China; ^4^ Department of Informatics, Obstetrics and Gynecology Hospital, Fudan University, Shanghai, China; ^5^ Department of Gastric Cancer & Soft Tissue Sarcoma Surgery, Fudan University Shanghai Cancer Center, Shanghai, China; ^6^ Ministry of Education Key Laboratory of Contemporary Anthropology and State Key Laboratory of Genetic Engineering, School of Life Sciences, Fudan University, Shanghai, China; ^7^ Fudan-Taizhou Institute of Health Sciences, Taizhou, Jiangsu, China; ^8^ Department of Biostatistics, Jiangsu Key Laboratory of Cancer Biomarkers, Prevention and Treatment, Collaborative Innovation Center For Cancer Personalized Medicine, The Key Laboratory of Modern Toxicology of Ministry of Education, School of Public Health, Nanjing Medical University, Nanjing, China

**Keywords:** gastric cancer, genetic susceptibility, miR-146a, polymorphism

## Abstract

The single nucleotide polymorphism (SNP) rs2910164 G>C within miR-146a has been reported that is associated with the increased risk of gastric cancer (GCa). However, the results are inconclusive, espicially among Asian populations, which probably due to small sample size in each single study. To validate this association and get a more precise estimation, we conducted a large GCa study including 1,125 cases and 1,196 controls in an eastern Chinese population. Our results showed that this SNP was not associated with GCa risk in either of the three genetic models [co-dominant model: CG vs. CC, odds ratio (OR) = 0.99, 95% confidence interval (95%CI): 0.83-1.19; GG vs. CC, OR = 1.03, 95%CI: 0.81-1.32; dominant model: (CG+GG) vs. CC, OR = 1.00, 95%CI = 0.84-1.19; recessive model: GG vs. (CG+CC), OR = 1.04, 95%CI = 0.83-1.29]. Stratified analysis by age, gender, smoking status, drinking status, or tumor location confirmed this non-significant association. In summary, these results suggest that the *miR-146a* SNP rs2910164 may not be a risk factor for GCa in this Chinese population. Larger and well-designed, preferably prospective studies are needed to further confirm our findings.

## INTRODUCTION

In China, Gastric cancer (GCa) is the second most common cancer, with an estimated 679,100 new GCa cases and 498,000 deaths in 2015, accounting for 15.8% of the cancer cases and 17.7% of cancer deaths, respectively [1]. However, the underlying mechanism of its carcinogenesis is still not fully understood. The environmental factors, such as toacco smoking, alcohol use, *helicobacter pylori* (*HP*) infection, as well as low-penetrance susceptibility genes are believed to be crucial in the etiology of GCa development [2, 3]. In addition, emerging genomic studies in recent years have identified a few genetic variants associated with GCa risk [4–8]; however, it is necessary to validate those previous reported genetic risk factors in the external populations.

miRNA, a small non-coding RNA molecule consisting of ~22 nucleotides, has crucial biological functions in post-transcriptional regulation of genes, as well as cell differentiation, proliferation, and apoptosis [9–12]. All these functions have critical roles in the development of cancer, including gastric cancer [13, 14]. Notebly, single nucleotide polymorphisms (SNPs) in miRNA genes could repress the efficiency of miRNA transcript processing [15]. The SNP rs2910164 within *microRNA-146a* (miR-146a) is placed in the passenger strand, of which the C allele probably causes hairpins mispaired [16] and thus, affect miR-146a maturing process [17]. This miRNA SNP has been extensively explored to cancer risk in scientific community [17–20]. The SNP rs2910164 G>C has been reported which is associated with the increased risk of GCa [21, 22]. However, the results are inconclusive, espicially among Asian populations [21, 23–25], which probably due to the moderate effect size or small sample size in each single study. Recently, an updated meta-analysis in this field suggested a correlation between this SNP and increased GCa risk [26]. However, due to insufficient sample size of each recruited study and potential heterogeneity among these study cohorts, the results of meta-analysis should be interpreted with caution. Therefore, we conducted a large GCa study of 1,125 cases and 1,196 controls in a well-established gastric cancer study cohort to validate this association in an eastern Chinese population.

## RESULTS

Table 1 described the population characteristics of this hospital-based case-control study, as reported previously [27]. Briefly, 1,125 GCa cases were averagely aged at 58.60±11.36 years, 71.1% male; 1,196 age and gender matched cancer-free controls were aged at 58.62 ± 11.75 years, and 69.1% were male. Age, gender, smoking status, as well as drinking status were further adjusted for the following multivariate analysis.

**Table 1:** Distributions of selected variables in gastric cancer cases and cancer-free controls

Variables	Cases N(%)	Controls N(%)	*P*^a^
All subjects	1,125(100)	1,196 (100)	
Age, yr			0.557
Range	21-86	22-86	
Mean^b^	58.60 ± 11.36	58.62 ± 11.75	
≤ 50	234 (20.8)	271 (22.7)	
51-60	383 (34.0)	384 (32.1)	
61-70	339 (30.1)	372 (31.1)	
>70	169 (15.0)	169 (14.1)	
Sex			0.282
Males	800 (71.1)	826 (69.1)	
Females	325 (28.9)	370 (30.9)	
Smoking status			<0.0001
Never	686 (61.0)	610 (51.0)	
Former	17 (1.5)	120 (10.0)	
Current	422 (37.5)	466 (39.0)	
Drinking status			0.008
Yes	270 (24.0)	345 (28.8)	
No	855 (76.0)	851 (71.2)	
Pack-years			<0.0001
0	686 (61.0)	610 (51.0)	
≤ 25 (mean)	227 (20.2)	355 (29.7)	
> 25 (mean)	212 (18.8)	231 (19.3)	
Tumor site			
GCA	305 (27.1)	—	
NGCA	820 (72.9)	—	

Abbreviations: GCA, gastric cardia adenocarcinoma; NGCA, non-gastric cardia adenocarcinoma

a Two-sided χ^2^ test for distributions between cases and controls.

b Age was described as mean ± SD.

Allele frequencies of the rs2910164 A>G SNP were listed by cases and controls in Table 2, as well as the association between this SNP and GCa risk. SNP rs2910164 showed a non-significant association with GCa risk in our study population [co-dominant genetic model: heterozygotes (CG) vs. wild-type (CC), odds ratio (OR) = 0.99, 95% confidence interval (95%CI): 0.83-1.19; homozygotes (GG) vs. CC, OR = 1.03, 95%CI: 0.81-1.32; dominant genetic model: (CG+GG) vs. CC, OR = 1.00, 95%CI = 0.84-1.19; recessive genetic model: GG vs. (CG+CC), OR = 1.04, 95%CI = 0.83-1.29]. Stratification analyses according to age, gender, smoking, drinking, and tumor location indicated consistent results (Table 3).

**Table 2:** Logistic regression analysis of associations between the genotypes of *miR146A* and gastric cancer risk

Variants	Genotypes	Cases (N=1,125)	Controls (N=1,196)	*P*^a^	Crude OR (95% CI)	*P*	Adjusted OR (95% CI)^b^	*P*^b^
Rs2910164								
	CC	397 (35.3)	420 (35.1)	0.946^c^	1.00		1.00	
	CG	536 (47.6)	577 (48.2)		0.98 (0.82-1.18)	0.850	0.99 (0.83-1.19)	0.922
	GG	192 (17.1)	199 (16.6)		1.02 (0.80-1.30)	0.868	1.03 (0.81-1.32)	0.801
	CG/GG	728 (64.7)	776 (64.9)	0.931^d^	0.99 (0.84-1.18)	0.931	1.00 (0.84-1.19)	0.988
Additive genetic model					1.01 (0.90-1.13)	0.930	1.01 (0.90-1.14)	0.853
	CC/CG	933 (82.9)	997 (83.4)		1.00		1.00	
	GG	192 (17.1)	199 (16.6)	0.783^e^	1.03 (0.83-1.28)	0.783	1.04 (0.83-1.29)	0.744

a Chi square test for genotype distributions between cases and controls

b Adjusted for age, sex, smoking and drinking status in logistic regression models

c for additive genetic models;

d for dominant genetic models;

e for recessive genetic models.

**Table 3:** Stratification analysis for associations between *miR146A* variant genotypes and gastric cancer risk

Variables	Rs2910164 (Cases/Controls)				Crude OR (95% CI)	*P*	Adjusted OR^a^ (95% CI)	*P*^a^
	CC		CG/GG					
	*N*	%	*N*	%				
Age, yr								
≤59 (median)	202/217	34.9/35.6	376/393	65.1/64.4	1.03 (0.81-1.30)	0.822	1.04 (0.82-1.32)	0.769
>59 (median)	195/203	35.6/34.6	352/383	64.4/65.4	0.96 (0.75-1.22)	0.723	0.95 (0.74-1.22)	0.677
Gender								
Males	291/284	36.4/34.4	509/542	63.6/65.6	0.92 (0.75-1.12)	0.401	0.93 (0.76-1.14)	0.496
Females	106/136	32.6/36.8	219/234	67.4/63.2	1.20 (0.88-1.64)	0.253	1.19 (0.86-1.63)	0.291
Smoking status								
Never	249/214	36.3/35.1	437/396	63.7/64.9	0.95 (0.76-1.19)	0.649	0.97 (0.77-1.22)	0.764
Former	6/39	35.3/32.5	11/81	64.7/67.5	0.88 (0.30-2.56)	0.819	1.10 (0.36-3.36)	0.872
Current	142/167	33.6/35.8	280/299	66.4/64.2	1.10 (0.84-1.45)	0.495	1.09 (0.82-1.44)	0.560
Drinking status								
Never	309/300	36.1/35.3	546/551	63.9/64.7	0.96 (0.79-1.17)	0.702	0.97 (0.80-1.19)	0.771
Ever	88/120	32.6/34.8	182/225	67.4/65.2	1.10 (0.79-1.55)	0.569	1.10 (0.79-1.55)	0.572
Pack-years								
0	249/214	36.3/35.1	437/396	63.7/64.9	0.95 (0.76-1.19)	0.649	0.96 (0.76-1.21)	0.726
≤25 (mean)	72/130	31.7/36.6	155/225	68.3/63.4	1.24 (0.87-1.77)	0.226	1.20 (0.84-1.73)	0.322
>25 (mean)	76/76	35.8/32.9	136/155	64.2/67.1	0.88 (0.59-1.30)	0.514	0.97 (0.64-1.46)	0.877
Tumor site								
GCA	110/420	36.1/35.1	195/776	63.9/64.9	0.96 (0.74-1.25)	0.756	0.96 (0.74-1.25)	0.752
NGCA	287/420	35.0/35.1	533/776	65.0/64.9	1.01 (0.83-1.21)	0.957	1.01 (0.84-1.22)	0.901

Abbreviations: GCA, gasric cardia adenocarcinoma; NGCA, non-gastric cardia adenocarcinoma

a Obtained in logistic regression models with adjustment for age, sex, smoking and drinking status

## DISCUSSION

Genetic susceptibility has been a research focus in cancer studies. Recently, *miR-146a* has drawn an increasing attention for its potential connection to several types of cancers, including gastric cancer. Several studies have indicated the common SNP rs2910164 in *miR-146a* as a moderate risk allele for gastric cancer [26, 28]. However, the results should be intepreted with causion, largely due to inadequate sample size in each independent study. Our study was performed with a relatively large sample size in a well-established gastric cancer study cohort among eastern Chinese population. This study showed an insignificant association, as well as among a series of subgroup analyses. The results suggest a considerably heterogeneous effect of this SNP among various cancer types. On the other hand, the observed association may be due to chance.

We acknowledge some limitations of the present study. First, although age, sex, smoking and drinking status, and tumor site were taken into consideration for subgroup analysis, other important risk factors, such as diet, microbial virulence, and HP infection, were missing in the study, which might also contribute to the etiology of GCa [29, 30]. Second, new classification of GCa tumor types, which was not available for the patients diagnosed years ago, is also important and may have an interaction effect with genetic variants on gastric cancer risk [31]. Third, the number of cases was largely reduced in the stratified analysis, which may lead to an insufficient statistical power.

In summary, these results suggest that the SNP rs2910164 of miR-146a may not be associated with the risk of GCa in this Chinese population. However, analysis of this SNP incorporating diet, *HP* infection status or Lauren classification probably provide an updated result.

## MATERIALS AND METHODS

### Study subjects

This study included patients who were recruited from our ongoing molecular epidemiology study of GCa, and the cases and controls were described previously [27, 32, 33]. Briefly, 1,125 unrelated ethnic Han Chinese patients with newly diagnosed and histopathologically confirmed primary gastric cardia adenocarcinoma and non-gastric cardia adenocarcinoma (NGCA) were recruited from Fudan University Shanghai Cancer Center (FUSCC) in Eastern China between January 2009 and March 2011. Patients other than histopathologically confirmed primary GCa were excluded. In addition, 1,196 age and sex-matched cancer-free ethnic Han Chinese controls were recruited from the Taizhou Longitudinal (TZL) study conducted at the same time period in Eastern China as described previously [34]. Blood samples from both GCa patients and cancer-free controls were provided by the tissue bank of FUSCC and the TZL study, respectively. All participants had signed a written informed consent for donating their biological samples to the tissue bank for scientific research. Demographic data and environmental exposure history of each participants were collected. The overall response rate was approximately 91% for cases and 90% for controls. This research protocol was approved by the FUSCC Institutional Ethics Review Board.

### SNP genotyping

According to a relevant protocol, we extracted genomic DNA from peripheral blood. The rs2910164 SNP was genotyped by the TaqMan assay with ABI7900HT real-time PCR system (Applied Biosystems) as reported previously [27]. Participants' status was unrevealed in the genotyping process. As recommend by the company, four negative controls (without DNA templates) and two duplicated samples were included in each 384-plate for the quality control. The assays were repeated for 5% of the samples, and the results were 100% concordant.

### Statistical methods

The χ^2^ test was used to assess differences in the distributions of demographic characteristics between cases and controls. The association between SNP and GCa risk was assessed by odds ratio (OR) and 95% confidence intervals (CIs) in heterozygous (CG vs. CC), homozygous (GG vs. CC), dominant (CG+GG vs. CC), recessive (GG vs. CG+CC), and additive (G vs. C) genetic models, respectively. OR values were calculated by both univariate and multivariate logistic regression models. Moreover, logistic regression tests for each genetic model were adjusted for age, sex, drinking and smoking status. Furthermore, the association between the *miR-146a* rs2910164 SNP and GCa risk was also stratified by age, sex, smoking or drinking status, and primary tumor site. All statistical process above was achieved by SAS Version 9.1 software (SAS Institute, Cary, NC, USA).
